# Impact of full field digital mammography diagnosis for female patients with breast cancer

**DOI:** 10.1097/MD.0000000000015175

**Published:** 2019-04-19

**Authors:** Tuan Wang, Jian-jun Shuai, Xing Li, Zhi Wen

**Affiliations:** a Department of Radiology, Affiliated Tumor Hospital of Xinjiang Medical University; b Department of Imaging Center, Traditional Chinese Medicine Hospital of Xinjiang Uyghur Autonomous Region; c Department of Nuclear Magnetic; d Department of Computed Tomography, Affiliated Tumor Hospital of Xinjiang Medical University, Urumqi, China.

**Keywords:** breast cancer, full field digital mammography, sensitivity, specificity

## Abstract

**Background:**

Previous clinical studies have reported that full field digital mammography (FFDM) can be used for diagnosis on breast cancer (BC) with promising outcome results. However, no study systematically investigates its diagnostic impact on female patients with BC. Thus, this systematic review will assess the accurate of FFDM diagnosis on BC.

**Methods:**

In this study, we will perform a comprehensive search strategy in the databases as follows: Cochrane Library, EMBASE, MEDILINE, PSYCINFO, Web of Science, Cumulative Index to Nursing and Allied Health Literature, Allied and Complementary Medicine Database, Chinese Biomedical Literature Database, China National Knowledge Infrastructure, VIP Information, and Wanfang Data from inception to February 28, 2019. All case-controlled studies exploring the impacts of FFDM diagnosis for patients BC will be fully considered for inclusion in this study. Two authors will independently scan the title and abstracts for relevance, and assess full texts for inclusion. They will also independently extract data and will assess methodological qualify for each included study by using Quality Assessment of Diagnostic Accuracy Studies (QUADAS-2) tool. RevMan V.5.3 software (London, UK) and Stata V.12.0 software (Texas, USA) will be used to pool the data and to conduct the meta-analysis.

**Results:**

The sensitivity, specificity, positive likelihood ratio, negative likelihood ratio, and diagnostic odds ratio of FFDM will be used to determine the diagnostic accuracy of FFDM for the diagnosis of patients with BC.

**Conclusion:**

Its findings will provide latest evidence for the diagnostic accuracy of FFDM in female patients with BC.

**Systematic review registration:**

PROSPERO CRD42019125338.

## 1 Introduction

Breast cancer (BC) is the most common gynecological cancers among female population around the world.^[1–3]^ It is also the most common cause of cancer death.^[4–6]^ The incidence and mortality rates have increased yearly worldwide.^[7–8]^ It has been estimated that about 63,960 cases were diagnosed and 266,120 cases are invasive cancer in 2018.^[9]^ In China, it is estimated that females account for 12.2% of all newly diagnosed BC, and 9.6% of all deaths from BC worldwide.^[10]^

It has been found that mammography, such as full field digital mammography (FFDM) can greatly reflect the fibro-glandular tissue in a women's breast.^[11–12]^ Thus, it is often used and is regarded as the gold standard imaging tool for BC screening among female population.^[6,13,14]^ Numerous clinical studies have reported to use FFDM for BC diagnosis and have achieved very promising outcome results.^[15–29]^ However, no study systematically has assessed its accuracy on patients with BC. Thus, in this study, we will systematically evaluate the diagnostic accuracy of FFDM for patients with BC.

## 2 Methods

### 2.1 Objective

This systematic review will aim to assess the value of FFDM in the diagnosis of female patients with BC.

### 2.2 Study registration

This study has been registered on PROSPERO CRD42019125338. It has been reported follow the guideline of Preferred Reporting Items for Systematic Reviews and Meta-Analysis Protocol (PRISMA-P) statement.^[30]^

### 2.3 Inclusion criteria for study selection

#### 2.3.1 Type of studies

This study will include case-controlled studies reporting the diagnostic accuracy of FFDM for the diagnosis of female patients with BC. However, we will exclude non-clinical studies, case report, case series, and non-controlled studies.

#### 2.3.2 Type of participants

In this study, the reports of female patients with histologically proven BC will be fully considered for inclusion.

#### 2.3.3 Type of index test

Index test: any forms of FFDM will be used to diagnose patients with BC. However, the combinations of FFMD with other diagnostic test will be excluded.

Reference test: patients with histologically proven BC only will be considered in the control group.

#### 2.3.4 Type of outcome measurements

The primary outcome measurements include sensitivity and specificity. The secondary outcome measurements consist of positive likelihood ratio, negative likelihood ratio, and diagnostic odds ratio.

### 2.4 Data sources and search strategy

#### 2.4.1 Electronic searches

The electronic databases of Cochrane Library, EMBASE, MEDILINE, PSYCINFO, Web of Science, Cumulative Index to Nursing and Allied Health Literature, Allied and Complementary Medicine Database, Chinese Biomedical Literature Database, China National Knowledge Infrastructure, VIP Information, and Wanfang Data will be searched from inception to February 28, 2019 without any language restrictions. The sample of search strategy for Cochrane Library is shown in Table 1. Identified search strategies will also applied to other electronic databases.

**Table 1:**
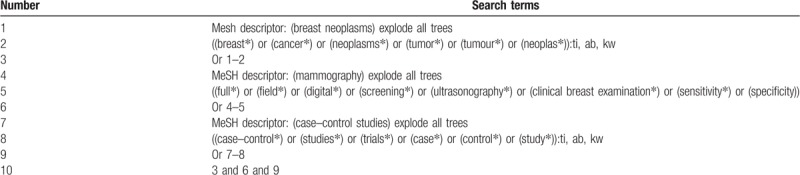
Search strategy used in Cochrane Library database.

#### 2.4.2 Other resources

Clinical registry and reference list of included studies and relevant reviews will also be searched.

### 2.5 Data collection and analysis

#### 2.5.1 Selection of studies

Endnote 7.0 software (Philadelphia, PA) will be used to manage the literature search records and remove the duplicated studies. Two authors will independently scan titles and abstracts initially according to the predefined eligibility criteria. Then, they will read full texts to further judge if they can meet the final eligibility criteria. The process of study selection will follow the PRISMA-P guidelines, and will be shown in PRISMA flowchart. Any divergences between 2 authors will be settled down by a third author.

#### 2.5.2 Data collection and management

Two authors will independently extract data by using pre-designed form for data extraction. Any disagreements regarding the data extraction between 2 authors will be solved by consulting a third author. The following information will be collected and relevant data will be extracted, including study characteristics, such as title, authors, year of publication, country; patient characteristics, such as race, age, sex, inclusion and exclusion criteria; details of diagnostic methods; study method, such as randomization, blinding, and concealment; outcome measurements, such as the number of true positives and negatives, false positives and negatives for each diagnostic test from each included study performance 2 × 2 tables.

#### 2.5.3 Dealing with missing data

If there are insufficient data, we will contact the primary authors to require those data. If those data are not achievable, we will only analyze the available data.

### 2.6 Methodological quality assessment

The methodological quality will be measured by Quality Assessment of Diagnostic Accuracy Studies (QUADAS-2) tool for each included study.^[31]^ This tool consists of 4 domains: patient selection, index test, reference standard, and flow and timing. Each domain is evaluated with risk of bias, which is judged by using signaling questions. The first 3 fields are also evaluated in terms of concerns regarding applicability. Two authors will independently assess the methodological quality for each study. Any disagreements will be resolved by discussion with a third author.

### 2.7 Statistical analysis

RevMan V.5.3 software and Stata V.12.0 software will be used to analyze the data and to carry out the meta-analysis. Data will be entered into Stata V.12.0 software and will plot estimates of sensitivity, specificity, positive likelihood ratio, negative likelihood ratio, and diagnostic odds ratio from the diagnostic 2 × 2 tables of primary studies. Descriptive statistics with 95% confidence intervals will be calculated for each primary study. Then, a descriptive forest plot will be derived. In addition, a summary receiver operating characteristic plot will also be performed.

#### 2.7.1 Assessment of heterogeneity

The degree of statistical heterogeneity will be identified by measured I^2^ statistic. If I^2^ ≤50%, low heterogeneity is considered. Otherwise, if I^2^ >50%, the significant heterogeneity will be regarded.

#### 2.7.2 Data synthesis

If heterogeneity is low (I^2^ ≤50%), data will be pooled, and meta-analysis will be conducted. If heterogeneity is significant (I^2^ >50%), data will be pooled according to the results of subgroup analysis. If the heterogeneity is still significant after subgroup analysis, data will not be pooled, and meta-analysis will not be performed directly. However, a bivariate random-effects regression approach will be utilized for summary of estimates of sensitivity and specificity.

#### 2.7.3 Subgroup analysis

Subgroup analysis will be carried out to detect any causes that may result in significant heterogeneity according to the different study characteristics, and patient characteristics.

#### 2.7.4 Sensitivity analysis

Sensitivity analysis will be conducted by eliminating the low methodological quality studies.

#### 2.7.5 Reporting bias

We will carry out funnel plots and associated regression tests to check if there are any publication biases.^[32]^

### 2.8 Ethics and dissemination

This study does not require research ethic, because it will not analyze individual patient data. The results of this study are expected to be published on peer-reviewed journals.

## 3 Discussion

This systematic review will first investigate the diagnostic accuracy of FFDM in female patients with BC by assessing the sensitivity, specificity, positive likelihood ratio, negative likelihood ratio, and diagnostic odds ratio. The results of this systematic review will provide a summary of the most recent evidence on the diagnostic accuracy of FFDM for BC. Its findings may also provide helpful evidence for the BC diagnosis and further researchers.

## Author contributions

**Conceptualization:** Tuan Wang, Xing Li, Zhi Wen.

**Data curation:** Tuan Wang, Jian-jun Shuai, Zhi Wen.

**Formal analysis:** Jian-jun Shuai, Xing Li.

**Funding acquisition:** Tuan Wang.

**Investigation:** Zhi Wen.

**Methodology:** Tuan Wang, Jian-jun Shuai, Xing Li.

**Project administration:** Tuan Wang, Zhi Wen.

**Resources:** Tuan Wang, Jian-jun Shuai, Xing Li.

**Software:** Tuan Wang, Jian-jun Shuai, Xing Li.

**Supervision:** Zhi Wen.

**Validation:** Jian-jun Shuai, Xing Li.

**Visualization:** Tuan Wang, Xing Li, Zhi Wen.

**Writing – original draft:** Tuan Wang, Jian-jun Shuai, Xing Li, Zhi Wen.

**Writing – review & editing:** Tuan Wang, Jian-jun Shuai, Xing Li, Zhi Wen.
